# Sperm whale long-range echolocation sounds revealed by ANTARES, a deep-sea neutrino telescope

**DOI:** 10.1038/srep45517

**Published:** 2017-04-12

**Authors:** M. André, A. Caballé, M. van der Schaar, A. Solsona, L. Houégnigan, S. Zaugg, A. M. Sánchez, J. V. Castell, M. Solé, F. Vila, D. Djokic, S. Adrián-Martínez, A. Albert, M. Anghinolfi, G. Anton, M. Ardid, J.-J. Aubert, T. Avgitas, B. Baret, J. Barrios-Martí, S. Basa, V. Bertin, S. Biagi, R. Bormuth, M. C. Bouwhuis, R. Bruijn, J. Brunner, J. Busto, A. Capone, L. Caramete, J. Carr, S. Celli, T. Chiarusi, M. Circella, A. Coleiro, R. Coniglione, H. Costantini, P. Coyle, A. Creusot, A. Deschamps, G. De Bonis, C. Distefano, I. Di Palma, C. Donzaud, D. Dornic, D. Drouhin, T. Eberl, I. El Bojaddaini, D. Elsässer, A. Enzenhöfer, K. Fehn, I. Felis, L. A. Fusco, S. Galatà, P. Gay, S. Geißelsöder, K. Geyer, V. Giordano, A. Gleixner, H. Glotin, R. Gracia-Ruiz, K. Graf, S. Hallmann, H. van Haren, A. J. Heijboer, Y. Hello, J. J. Hernandez-Rey, J. Hößl, J. Hofestädt, C. Hugon, G. Illuminati, C. W. James, M. de Jong, M. Jongen, M. Kadler, O. Kalekin, U. Katz, D. Kießling, A. Kouchner, M. Kreter, I. Kreykenbohm, V. Kulikovskiy, C. Lachaud, R. Lahmann, D. Lefèvre, E. Leonora, S. Loucatos, M. Marcelin, A. Margiotta, A. Marinelli, J. A. Martínez-Mora, A. Mathieu, K. Melis, T. Michael, P. Migliozzi, A. Moussa, C. Mueller, E. Nezri, G. E. Păvălaş, C. Pellegrino, C. Perrina, P. Piattelli, V. Popa, T. Pradier, C. Racca, G. Riccobene, K. Roensch, M. Saldaña, D. F. E. Samtleben, M. Sanguineti, P. Sapienza, J. Schnabel, F. Schüssler, T. Seitz, C. Sieger, M. Spurio, Th. Stolarczyk, A. Sánchez-Losa, M. Taiuti, A. Trovato, M. Tselengidou, D. Turpin, C. Tönnis, B. Vallage, C. Vallée, V. Van Elewyck, D. Vivolo, S. Wagner, J. Wilms, J. D. Zornoza, J. Zuñiga

**Affiliations:** 1 Technical University of Catalonia, BarcelonaTech (UPC), Laboratory of Applied Bioacoustics (LAB), Rambla Exposició, 24, 08800 Vilanova i la Geltru, Barcelona, Spain; 2 Institut d’Investigació per a la Gestió Integrada de les Zones Costaneres (IGIC) - Universitat Politècnica de Valencia, C/Paranimf 1, 46730 Gandia, Spain; 3 GRPHE - Université de Haute Alsace - Institut universitaire de technologie de Colmar, 34 rue du Grillenbreit BP 50568 – 68008, Colmar, France; 4 INFN - Sezione di Genova, Via Dodecaneso 33, 16146 Genova, Italy; 5 Friedrich-Alexander-Universitat Erlangen-Nurnberg, Erlangen Centre for Astroparticle Physics, Erwin-Rommel-Str. 1, 91058 Erlangen, Germany; 6 Aix-Marseille Université, CNRS/IN2P3, CPPM UMR 7346, 13288 Marseille, France; 7 APC, Université Paris Diderot, CNRS/IN2P3, CEA/IRFU, Observatoire de Paris, Sorbonne Paris Cité, 75205 Paris, France; 8 IFIC - Institut de Física Corpuscular (CSIC – Universitat de València) c/Catedrático José Beltran, 2 E-46980 Paterna, Valencia, Spain; 9 LAM - Laboratoire d’Astrophysique de Marseille, Pôle de l’Etoile Site de Château-Gombert, rue Frédéric Joliot-Curie 38, 13388 Marseille Cédex 13, France; 10 INFN - Laboratori Nazionali del Sud (LNS), Via S. So a 62, 95123 Catania, Italy; 11 Nikhef, Science Park, Amsterdam, The Netherlands; 12 Huygens-Kamerlingh Onnes Laboratorium, Universiteit Leiden, The Netherlands; 13 Universiteit van Amsterdam, Instituut voor Hoge-Energie Fysica, Science Park 105, 1098 XG Amsterdam, The Netherlands; 14 INFN -Sezione di Roma, P.le Aldo Moro 2, 00185 Roma, Italy; 15 Dipartimento di Fisica dell’Universita La Sapienza, P.le Aldo Moro 2, 00185 Roma, Italy; 16 Institute for Space Science, RO-077125 Bucharest, Magurele, Romania; 17 INFN - Sezione di Bologna, Viale Berti-Pichat 6/2, 40127 Bologna, Italy; 18 INFN - Sezione di Bari, Via E. Orabona 4, 70126 Bari, Italy; 19 Geoazur, UCA, CNRS, IRD, Observatoire de la Côte d’Azur, Sophia Antipolis, France; 20 Univ. Paris-Sud, 91405 Orsay Cedex, France; 21 University Mohammed I, Laboratory of Physics of Matter and Radiations, B.P.717, Oujda 6000, Morocco; 22 Institut für Theoretische Physik und Astrophysik, Universität Wärzburg, Emil-Fischer Str. 31, 97074 Würzburg, Germany; 23 Dipartimento di Fisica e Astronomia dell’Universita, Viale Berti Pichat 6/2, 40127 Bologna, Italy; 24 Laboratoire de Physique Corpusculaire, Clermont Université, Université Blaise Pascal, CNRS/IN2P3, BP 10448, F-63000 Clermont-Ferrand, France; 25 INFN - Sezione di Catania, Viale Andrea Doria 6, 95125 Catania, Italy; 26 LSIS, Aix Marseille Université CNRS ENSAM LSIS UMR 7296 13397 Marseille, France; 27 Royal Netherlands Institute for Sea Research (NIOZ), Landsdiep 4, 1797 SZ ‘t Horntje (Texel), The Netherlands; 28 Dipartimento di Fisica dell’Universita, Via Dodecaneso 33, 16146 Genova, Italy; 29 Dr. Remeis-Sternwarte and ECAP, Universitat Erlangen-Nurnberg, Sternwartstr. 7, 96049 Bamberg, Germany; 30 Moscow State University, Skobeltsyn Institute of Nuclear Physics, Leninskie gory, 119991 Moscow, Russia; 31 Mediterranean Institute of Oceanography (MIO), Aix-Marseille University, 13288, Marseille, Cédex 9, France; 32 Dipartimento di Fisica ed Astronomia dell’Universita, Viale Andrea Doria 6, 95125 Catania, Italy; 33 Direction des Sciences de la Matière - Institut de recherche sur les lois fondamentales de l’Univers - Service de Physique des Particules, CEA Saclay, 91191 Gif-sur-Yvette Cédex, France; 34 INFN - Sezione di Pisa, Largo B. Pontecorvo 3, 56127 Pisa, Italy; 35 Dipartimento di Fisica dell’Universita, Largo B. Pontecorvo 3, 56127 Pisa, Italy; 36 INFN -Sezione di Napoli, Via Cintia 80126 Napoli, Italy; 37 Université de Strasbourg, IPHC, 23 rue du Loess 67037 Strasbourg, France; 38 Dipartimento di Fisica dell’Universita Federico II di Napoli, Via Cintia 80126, Napoli, Italy; 39 Université de Toulon CNRS LSIS UMR 7296 83957 La Garde, France; 40 Institut universitaire de France, 75005 Paris, France; 41 Université du Sud Toulon-Var, 83957 CNRS-INSU/IRD UM 110, La Garde Cédex, France; 42 CNRS, UMR7178, 67037 Strasbourg, France

## Abstract

Despite dedicated research has been carried out to adequately map the distribution of the sperm whale in the Mediterranean Sea, unlike other regions of the world, the species population status is still presently uncertain. The analysis of two years of continuous acoustic data provided by the ANTARES neutrino telescope revealed the year-round presence of sperm whales in the Ligurian Sea, probably associated with the availability of cephalopods in the region. The presence of the Ligurian Sea sperm whales was demonstrated through the real-time analysis of audio data streamed from a cabled-to-shore deep-sea observatory that allowed the hourly tracking of their long-range echolocation behaviour on the Internet. Interestingly, the same acoustic analysis indicated that the occurrence of surface shipping noise would apparently not condition the foraging behaviour of the sperm whale in the area, since shipping noise was almost always present when sperm whales were acoustically detected. The continuous presence of the sperm whale in the region confirms the ecological value of the Ligurian sea and the importance of ANTARES to help monitoring its ecosystems.

The last decade has seen tremendous progress in the construction of undersea neutrino observatories. These telescopes rely on the detection of Cherenkov light emitted along the path of charged particles produced in neutrino interactions^1^,^2^. When located under the sea, neutrino telescopes are also multidisciplinary observatories that have the potential to provide a new level of understanding of the marine ecosystems at great depths^3^.

The ANTARES (Astronomy with a Neutrino Telescope and Abyss environmental RESearch) experiment is the largest neutrino telescope presently operating in the Northern Hemisphere^1^. It is located in the deep Mediterranean Sea at a depth of 2,475 m. In addition to light sensors, the ANTARES infrastructure also incorporates the AMADEUS (ANTARES Modules for Acoustic DEtection Under the Sea^2^), system. This system comprises acoustic sensors (hydrophones) aiming at evaluating the potential for detection of ultra-high-energy neutrinos through the pressure waves they produce when interacting in water. The system of ANTARES comprises many acoustic hydrophones, designed to perform measurements of acoustic backgrounds in the deep sea.

Most oceanographic instruments moored on the seafloor are not connected with the surface: they have to run on batteries and store data locally, implying that scientists only have access to their data after the recovery of the instrument^3^. Cabled observatories such as ANTARES (France), JAMSTEC (Japan), NEPTUNE (Canada) and OBSEA (Spain), remove these restrictions by providing not only electrical power and real-time data acquisition, but also the possibility for scientists to interact directly with their sensors. They can control them and monitor the environment in reaction to the observation of peculiar events. New real-time opportunities are therefore given to marine sciences and geosciences: listening to marine organisms, observing bioluminescent creatures^4^, studying global change^5^ and its consequence on marine circulation and biodiversity.

Understanding the link between natural and anthropogenic processes is indeed essential for predicting the magnitude and impact of future changes of the natural balance of the oceans. Amongst this wide variety of changes, the next decades will see increasing levels of offshore industrial development, and this will almost certainly lead to increased amounts of noise pollution in the oceans.

Noise pollution in the marine environment is an emerging but serious concern to science and society^6^. Its implications are less well understood than other global threats and, until recently, largely undetectable to everyone but the expert. In addition, the assessment of the acoustic impact of artificial sounds in the sea is not a trivial task, certainly because there is a lack of information on how marine organisms process and analyse sounds and how relevant these sounds are for the balance and development of their populations^7^. Organisms that are exposed to sound can be adversely affected both on a short timescale (acute effect) and on a long timescale (permanent or chronic effects). These adverse effects can be widespread and the European Commission decided in September 2010, under the Marine Strategy Framework Directive (MSFD) that two indicators for underwater noise be used in describing ocean Good Environmental Status^6^.

As an essential step reaching Good Environmental Status, European Member States were asked to establish monitoring programmes for assessment, enabling the state of the marine waters concerned to be evaluated on a regular basis. The MSFD comes with criteria and methodological standards on GES of marine waters (Commission Decision 2010/477/EU), including two indicators on noise (Descriptor 11, Noise/Energy): Low and mid frequency impulsive sounds as well as continuous low frequency sound (ambient noise)^6^.

The need of including noise indicators in the MSFD is supported by recent findings, which patently showed that an exposure to low frequency sounds would trigger massive acoustic trauma in non-hearing specialists like cephalopods^8^,^9^,^10^. If we add that the negative consequences of a short or long term exposure to artificial sounds may not be immediately detected, one can understand how challenging it is to obtain objective data allowing an efficient control of the effects of anthropogenic sound in the sea.

Definitive studies on the response of marine mammals to anthropogenic sound are typically hampered by the short time spent at the surface and the deep-diving lifestyle of many vocalising species. Implemented within the framework of the European Sea-Floor Observatory Network of Excellence (ESONET) in 2007, the “Listen to the Deep Ocean Environment (LIDO, http://listentothedeep.com)” programme has developed and applied techniques for continuous noise measurement and passive acoustic monitoring (PAM) to world-wide cabled deep sea platforms and moored stations, representing the first and only PAM system available online (http://listentothedeep.com, ref. 11). The combination of this real-time data management and passive acoustic monitoring with the use of the latest technological developments in underwater neutrino acoustic detection has provided the scientific community with a hitherto non-available technology to reveal the daily behaviour of deep-sea marine organisms, therefore opening for the first time Internet access to deep ocean sound information.

The sperm whale (*Physeter macrocephalus*) is a successful deep-sea predator with a worldwide distribution. Sperm whales consume a wide diversity of prey: although squids appear to be their primary resource, fishes also are important in certain locations. It is largely accepted that sperm whales forage during deep dives that routinely exceed a depth of 400 m, sometimes reaching more than 2,000 m, and 30–40 min duration^12^. Their need of individually ingesting one ton of squid per day on average leads them to rarely remain in the same region for long, but to migrate year-round over large distances in search of food^12^.

While foraging, sperm whales produce short duration sound signals named clicks. *Usual clicks* are produced in a 10 Hz–30 kHz frequency band, at a rate defining an inter-click interval (ICI) that varies from 0.5 to 2.0 seconds during descent from the surface until the whale begins its ascent to the surface. There is evidence that usual clicks produced during foraging dives are directional, with an intense, forward-directed beam, presenting levels as high as 236 dB re 1 μPa at 1 m^13^. Their off-axis low frequency components can be detected up to a distance of 15 km in sea state 3. Recent data on sperm whale sounds suggested that usual clicks are appropriate for echolocation on these low-reflecting targets, thus providing support that sperm whales typically spend more than 80% of their time immersed in foraging processes^13^,^14^,^15^. In addition to usual clicks, sperm whales produce *creak clicks* during foraging that have properties equivalent to signals in buzzes, the terminating pulse trains known from echolocating bats during prey capture^15^. The frequency content of an on-axis creak click resembles that of a P1 pulse of on-axis usual clicks, which validates the same speculations for a match between the frequency content and backscatter of prey items in the meso- and bathypelagic ecosystem. Hence, creak clicks are just as suited for echolocation as are usual clicks, but the reduced output and high repetition rate suggest a shorter sonar range. The directional nature and the reduced ASL on-axis, compared with usual clicks, of 185–205 dB//1 μPa rms also make creak clicks hard to detect on a sufficient number of hydrophones to allow for localization of the source and subsequent derivation of source parameters^15^.

In this paper, we present the analysis of 2 years of uninterrupted acoustic data collected by the ANTARES telescope and automatically processed in real-time by the LIDO software package.: one year of data (2011) was used for training which results were then used for classification in the second year (2012). This allowed the training of classifiers that revealed and displayed directly on the Internet the daily long-range echolocation behaviour of the sperm whale in the deep Ligurian Sea, and a first year-round study of the presence and potential effects of shipping noise on this particular species.

## Results

Data was processed in consecutive data segments of 16.8 seconds length. Acoustic features were identified and classified for each segment, with 1323397 analyzed segments in total for the second year. Presence of a class was then evaluated per segment. In the following, SW, UC, and IS stand for the sperm whale, ultrasonic cetacean and shipping classes. When they are followed by a C (SWC, UCC, ISC) they describe the output of the GMM classifier as explained in the Methods section. When they are followed by an I (SWI, UCI, ISI) they represent the smoothed number of classified impulses in an analysed data segment. When they are followed by a P (SWP, ISP) they represent the output of the GLM presence model.

### Sperm whale presence model

A generalized linear model was fitted with response variable sperm whale presence and as independent variables the log transformed SWI (SWI’) and ISI (ISI’). The interaction between these two variables was also included. The noise level SPL and the log transformed UCI were initially tried as covariates but were not found to significantly model animal presence. The explained variance was 53%. As shown in Table 1, both SWI’ and the interaction SWI’ * ISI’ were significant while ISI’ by itself did not have a significant influence. The 95% confidence interval of the number of segments with sperm whale presence (SWP) was [115.14, 142.9] corresponding to 12% and 15% of the total number of segments analysed.


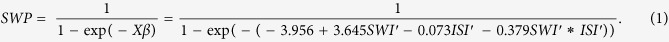


### Impulse ship presence model

Impulsive shipping noise presence (ISP) was modelled by a GLM using the information in the log transformed ISI, SWI and UCI. As for the sperm whale model, SPL did not have a significant influence. The explained deviation of the model was 55%. As shown in Table 2, the interaction SWI’ * ISI’ was included in the model. The 95% confidence interval of the number of segments with ISP was [367.5, 403.7], corresponding to between 38% and 42% of the total number of segments analysed.

### Missing data

Several gaps in time were found, mostly caused by shipping noise presence: the noise produced by e.g., a ferry that passed by twice a day occasionally required shutting down the acoustic data acquisition due to system saturation. Therefore, the number of missing segments increased between 01:00–02:00 and 20:00–21:00 UTC with the highest percentage of missing data at 02:00 UTC (28% of the segments missing). Nonetheless, all daily hours were well represented in the recorded data. In order to allow comparison between hours of the day, the estimates of the hourly SWP and ISP were normalized with the percentage of missing data in each hour. Impulses were detected in more than half of the total number of segments, with maximum rates between 19:00 and 03:00 UTC.

### Sperm whale and shipping presence recorded by the acoustic system of ANTARES during 2012

The number of segments with sperm whales and shipping impulses was estimated for each month of 2012 (Table 3). All the segments processed in each month were evaluated by the SWP model and the ISP model. The resultant predictions of each class were normalised to the proportion of missing data. Shipping presence was more common than sperm whale presence: 29% versus 15% of the time respectively.

Most importantly, Table 3 shows the continuous presence of sperm whales in the vicinity of ANTARES and an increased presence in summer.

**Figure 1:**
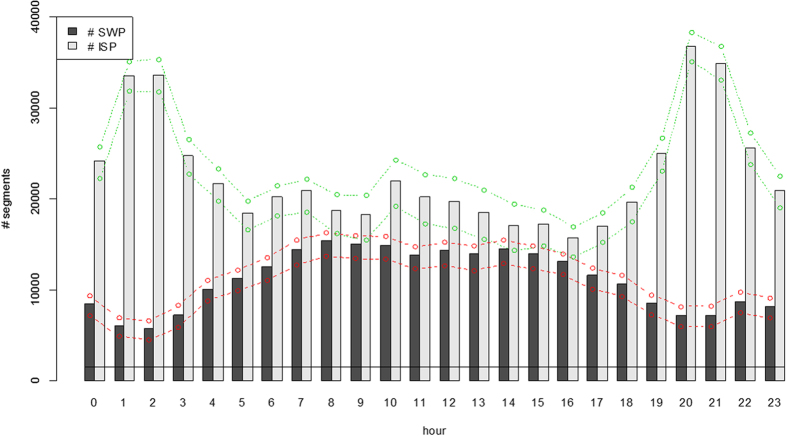
Number of segments with sperm whales and shipping impulse presence per hour in 2012. Sperm whale acoustic presence was higher during the daylight hours, whereas at night shipping impulses were more prominent. The red and green lines show the confidence interval at 95% for the SWP and ISP predictions, respectively, and the black line the sum of SWP predictions if the sperm whale model was evaluated with SWI = 0.

Shipping and sperm whale acoustic presence had contrasting behaviour during the day (Fig. 1). Although both classes contained many segments with presence over all 24 hours of the day, ISP had higher estimated values at night and SWP was larger during daylight hours. In terms of ISP, there were two apparent peaks at 01:00 and 20:00 UTC. Both corresponded to the regular passage of a ferry during those hours. The SWP predictions presented significant differences (Wilcoxon paired test: p-value < 0.001) over daylight hours (from 06:00 to 18:00 UTC) and night (from 18:00 to 06:00 UTC). In all twelve months of 2012 the SWP rate observed in daylight hours was larger than the rate at night. In Fig. 1, a 95% confidence interval is shown for the SWP predictions (red line) and ISP predictions (green line). The apparent diel shape for sperm whale presence anticorrelated with the shipping presence visible in Fig. 1 will be analyzed in detail at the discussion section below.

### Sperm whale presence as function of noise level

**Figure 2:**
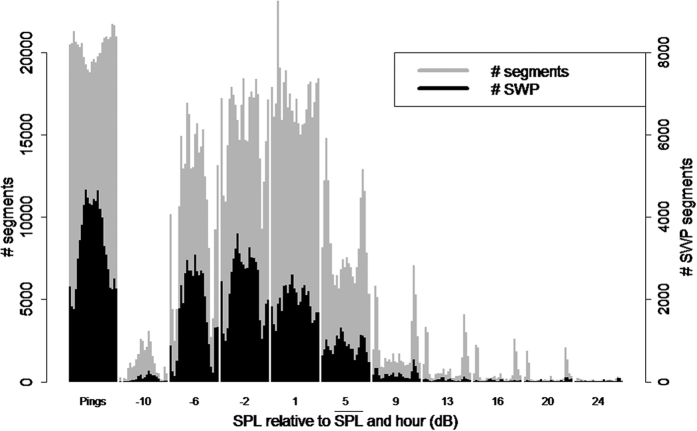
Number of recorded segments (grey, left y-axis) and segments with sperm whale acoustic presence (black, right y-axis) as function of hour and noise level (relative to SPL and hour). SPL were divided and rounded in ten bins. Each SPL features 24 bars corresponding to 24 hours.

The sperm whale hourly acoustic presence for different levels of noise is presented in Fig. 2. At each SPL, 24 bars corresponding to each hour of the day were drawn, showing the total recorded segments and the segments with sperm whale presence. As explained in the methods section, the SPL was always high in segments that contained pings from the line localisation echo sounders. To avoid mixing these segments with segments that had increased SPL due to a continuous noise source, the segments with pings are shown separately in the first group of bars.

The grey bars (Fig. 2) show differences according to the SPL. At lower levels, many more segments are processed in daylight hours than in the night. As the noise level increases, the tendency is inverted, with a larger quantity of segments being processed at night (with two local maxima at the beginning and at the end of the day). The distribution of the total number of segments also exhibits a long right tail with many detected impulses at high SPL. These are likely produced by the same ships that also caused the increase in the noise.

Sperm whales were acoustically detected during all hours of the day regardless of noise levels. The hourly SWP distribution, with a peak around midday, is present at SPL between −6 dB and 5 dB, and in the large dataset with the pings. When the relative SPL were over 9 dB very few sperm whales (or impulses in general) were detected and the hourly pattern of SWP (Fig. 2) is not visible anymore.

## Discussion

### Presence model

The training data used to fit the model contained some segments with false negative observations when there were actually sperm whale impulses. These impulses were either (1) too faint to be picked up at the detection stage or (2) the classifier did not recognize these impulses as being SWC. This is reflected in Table 4, where even when no sperm whales are detected (SWI = 0), the non-zero intercept results in a prediction of presence of about 0.02. The assumption that sperm whales can be present at any time (i.e. hour) at ANTARES throughout the year has been confirmed through human observation over the last three years.

Occasionally, when a high number of shipping impulses were produced, the classifier may have overestimated SWI by generating false positives. Shipping can produce hundreds of impulses in a single segment and even a small false positive rate may produce a significant SWI. This is reflected in the negative coefficient −0.379 for the SWI’ and ISI’ product, which lowers the influence caused by false positives during ISI detections.

One problem that the model in eq. (2) cannot take into account is false negatives in the detection and classification phases due to masking of sperm whale sonar. While some information from the SPL measurements is included in the model through ISI (a high number of shipping impulses indicates a nearby ship, which usually produces high noise levels), SPL itself had no significance. When all sperm whale echolocation signals are masked to the point that it is not even possible to manually establish acoustic presence, then no training set can be created that takes into account this kind of false negative.

### Reasons for divergence between shipping and sperm whale diel behaviour

The contrasting behaviour between the presence of ISI and sperm whales does not necessarily mean that sperm whales were avoiding high levels of noise caused by ships. Four different hypotheses could be posed: (1) sperm whales are more often present at daylight hours independently of shipping appearance; (2) sperm whales are present at least as often at night as at daylight hours; due to shipping occurring more at night: (3) sperm whale echolocation signals are misclassified as shipping at night but not at daylight hours; (4) sperm whale echolocation signals are masked by shipping impulses at night. Note that only hypothesis (1) and (2) are mutually exclusive.

**Figure 3:**
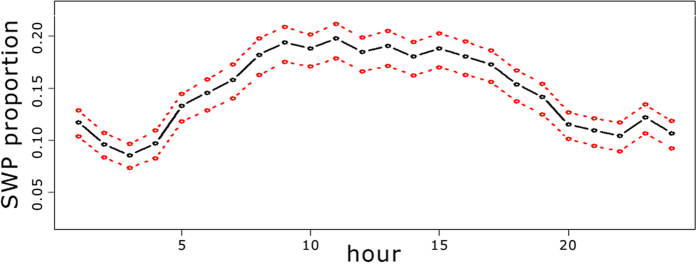
Proportion of sperm whale acoustic presence predictions considering only the segments with relative SPL lower than 1. A total of 917,233 segments were processed confirming a maximum at midday. The red line shows the confidence interval at 95%.

By taking a look at Fig. 1, we could presume that hypothesis (1) is the most probable one: there is a significant difference of sperm whale acoustic presence between night and day hours. However, this assumption cannot be proven with the results presented above. It is not clear if the daily presence is due to what is indicated in hypothesis (3) or in hypothesis (4), which could then lead to hypothesis (2) as the real situation. To have a better idea of which hypotheses are more probable and how these could affect the results, two additional arguments are introduced:

- The number of data segments that contained a high relative SPL (more than 7 dB over 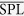) but did not contain an ANTARES localisation signal was less than 4% of the total number of segments. Thus, the masking of impulses did not occur often enough to drastically change the obtained results. When SPL was low enough to avoid masking, about 13.25% of the analysed segments contained sperm whales (the average ratio between black and grey bars in Fig. 1). If we suppose a similar ratio when the SPL was high (as per hypothesis 4) then the error in sperm whale presence estimation due to masking would not be larger than 600 segments for each hour or less than 8% of the segments with sperm whale presence in Fig. 1. In that case, even if hypothesis (4) is true, then it would not significantly affect the results.
- An hourly behaviour analysis was reproduced with only the noise levels where no masking was found. For instance, relative SPL between −10 to 1 dB were rarely affected by high noise level events (Fig. 2). Taking into account only segments in this SPL range, the ratio between the sum of SWP predictions and the total number of segments processed during each hour of the day was computed (Fig. 3).

At least two conclusions can be drawn from Fig. 3: (I) when no masking is produced the pattern seen in Fig. 3 seems consistent with the result obtained in Fig. 1. Hence, even if hypothesis (4) were true, when segments containing masking are discarded, providing more confidence in the reliability of the model, the conclusions seem to be in agreement with what was presented for the whole data set. (II) If we rely on the validity of the model, accurately estimating false positives in SWP and ISP from the training data, and we suppose that masking is due to high levels of noise instead of impulse ship presence, then the plot in Fig. 3 that includes all segments without masking should give the most reliable result. Although the hourly pattern could change slightly, it seems strong enough to indicate significant differences in the sperm whale acoustic presence during the different hours of the day.

### Discussion and conclusions in the framework of this study

The sperm whale is one of eight common cetacean species in the western Mediterranean Sea, and one of only two great whales encountered in the Mediterranean Sea^16^,^17^,^18^,^19^,^20^,^21^,^22^. Social groups have occasionally been reported in the recent past^17^,^18^, although schools of more than 15 individual animals were observed in the 1950s^20^. Genetic and observational evidence support the notion that the Mediterranean sperm whales constitute an isolated population, separated from the Atlantic stock. Robust information is lacking on their population status but their number would be in the hundreds rather than thousands and current densities appear to be much lower than those reported in the 1950s suggesting that a special attention should be devoted to the conservation status of this population^22^. By-catches linked to driftnet^23^, although illegal in the Mediterranean since 2002, have still been reported in recent times, while ship strikes and disturbance from maritime traffic represent a constant threat to this population^22^. This makes it vitally important to carefully monitor these potential sources of anthropogenic impact and to gather information on the current sperm whale distribution and behaviour to take conservation actions.

The analysis of two years of continuous acoustic data provided by the ANTARES neutrino telescope revealed the year-round presence of sperm whales in the Ligurian Sea, probably associated to the availability of their main prey in the region. Although creak clicks could not be detected at ANTARES probably due to distance, directionality and masking potential from other sources, the continuous presence of long-range echolocation sounds over time suggests that the sperm whales are foraging in the area. Several authors have previously noticed a link between sperm whale density and primary production^24^. While most of the Mediterranean is considered an oligotrophic sea, the western Ligurian Sea is characterised by high levels of primary productivity, caused by the interplay of climatic, oceanographic and physiographic factors^25^,^26^. A dominant cyclonic (counterclockwise) current, flowing north along Corsica and Tuscany and thence hugging the coast of Liguria and mainland France in a westerly direction, creates a permanent frontal system, which separates coastal and offshore waters^27^. Intense biological activity is generated along this water mass boundary by the enhanced productivity and retention associated with this frontal system^28^. Indeed, sperm whale vocalisations recorded at ANTARES are compatible with foraging behaviour, thus presumably indicating the availability of cephalopods – the sperm whale favourite diet - in the Ligurian Sea region, not only in summer but all year round.

**Figure 4:**
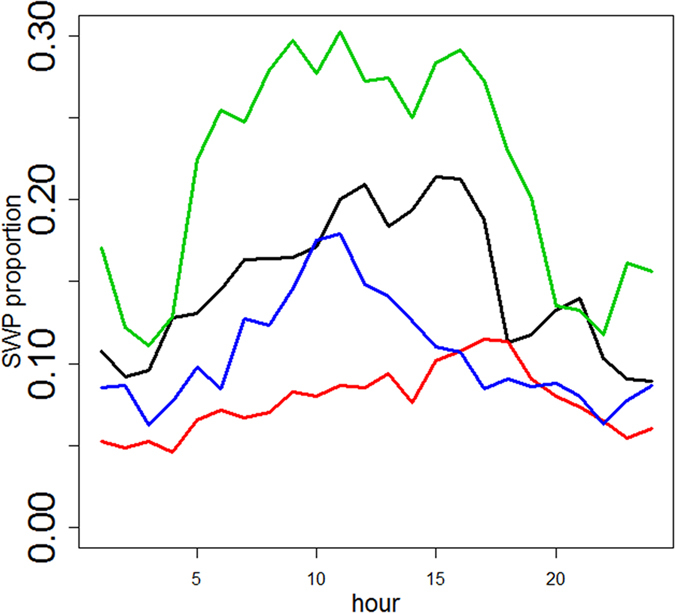
Proportion of sperm whale presence predictions per season (black line: winter, red line: spring, green line: summer and blue line: autumn) considering only the segments with relative SPL lower than 1.

The diel variation shown here in the feeding activity of the sperm whales in this region is probably also of relevance. Note that no seasonal variation was found in the detection of long-range echolocation sounds of the sperm whales (see Fig. 4). Diel patterns in behaviour have been documented for many marine mammals. For some whales, diel patterns are generally related to the timing of feeding, and hence should be appropriately related to environmental conditions, and food availability. Sperm whales feed mainly on mesopelagic and bathypelagic squid, such as squid from the families Gonatid, Histioteuthid and Onychoteuthid^29^. Some of these squid species, such as several species of *Gonatus*, conduct diel vertical migrations^30^, which may affect the foraging behaviour of sperm whales. However, the present results of increased sperm whale acoustic detection during daytime are in contrast with the findings by Matsushita^31^ who proposed that the sperm whales in the Antarctic feed mainly at night when the squid rise to the sea surface. This conclusion was made after examination of stomach contents of whales caught early in the morning and at night, whose size was greater than that of those caught during the day.

Although the results presented here suggest foraging diel behaviour, it must be taken into account that the detection range of sperm whale signals is limited at around 15 km from the ANTARES telescope, not descarding that sperm whale preys may be moving away from the area (and sperm whales may consequently feed outside Antares acoustic range), nor that sperm whales can remain quiet for some time when gathered at the surface. Nevertheless, the hitherto unknown continuous presence of the Ligurian Sea sperm whales was demonstrated for the first time through the real-time analysis of audio data streamed from a cabled-to-shore deep-sea observatory. The unprecedented use of this technique and infrastructures allowed the hourly tracking of their long-range echolocation sounds on the internet, revealing a probable diel presence pattern: the sperm whales would mostly feed on cephalopods during daylight off Toulon coastlines. Whether this behaviour can be related to their prey vertical migration or simply to the absence of prey at certain times of the day, is still under study. Most interestingly, the same acoustic analysis revealed that the influence of surface shipping noise doesn’t seem to condition the long-range echolocation behaviour of sperm whales in the area, since shipping noise was almost always present when sperm whales were detected. And although ship noise challenged the classification performance, sperm whales sounds were always detected at any time of the day. This continuous presence in the region confirms the ecological value of the Ligurian sea and the importance of ANTARES to help monitor its ecosystems. However, the high level of noise that marine fauna is continuously exposed to might represent in the medium term a threat to its conservation and efforts should be conducted to mitigate its effects, especially on a species like the sperm whale whose populations may be declining in the Mediterranean Sea.

## Methods

### The Acoustic Setup

**Figure 5:**
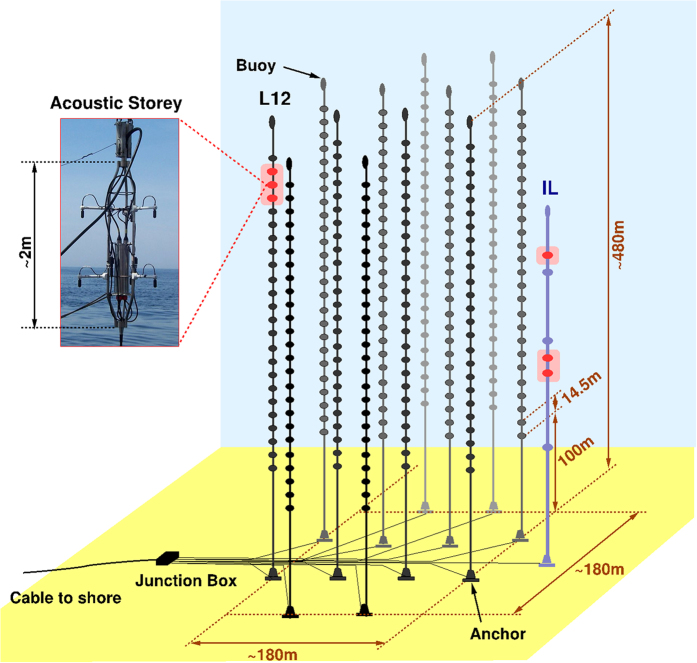
A sketch of the ANTARES detector. The six AMADEUS acoustic storeys are highlighted in pink-red (see text for details).

The AMADEUS acoustic detection equipment is designed to perform background measurements over a period of several years in an arrangement of acoustic sensors allowing studies at different temporal scales. ANTARES comprises 12 vertical structures, the detection lines, each one holding up to 25 storeys that are arranged at equal distances of 14.5 m along the lines^2^. Standard storeys contain sensors for the detection of Cherenkov light. Each line is anchored to the sea floor and held taut by a submerged buoy. A 13th line, called the Instrumentation Line (IL), is equipped with instruments for monitoring the environment. It holds six storeys. The acoustic sensor clusters of AMADEUS are distributed over the ANTARES detector with distances of 200 m horizontally and ranging from 10 m to 100 m vertically (Fig. 5). In total, 2 (out of 13) ANTARES lines are equipped with acoustic detection devices to form 6 so-called *acoustic storeys* representing the clusters of sensors, allowing position reconstruction of acoustic point sources for the frequency range from about 1 to 125 kHz^2^. Channel 15 in storey 24 (middle red dot hydrophone, L12, Fig. 5) was chosen for the analysis described in this paper because of its very low self-noise. Sampling rate was 250 kHz, hydrophone sensitivity −170 dB re 1 V/μPa, gain 10 dB, quantization +2 V.

### The LIDO classification process

The automated detection and classification of impulsive sounds in the marine environment is challenging. The intensity and the spectrum of the background noise is generally variable over periods of hours or even minutes due to changes in sea state or local anthropogenic activity, particularly shipping, which reduces the ability to detect weak impulses with a reasonably small false detection rate. Impulses are short and often only cover a fraction of the data stream. They must be automatically identified with sufficient precision by a detection stage. The acoustic features, which are extracted from each impulse, must give consistent values even under variations of the intensity and the spectrum of the background noise and the occurrence of interfering sounds.

**Figure 6:**
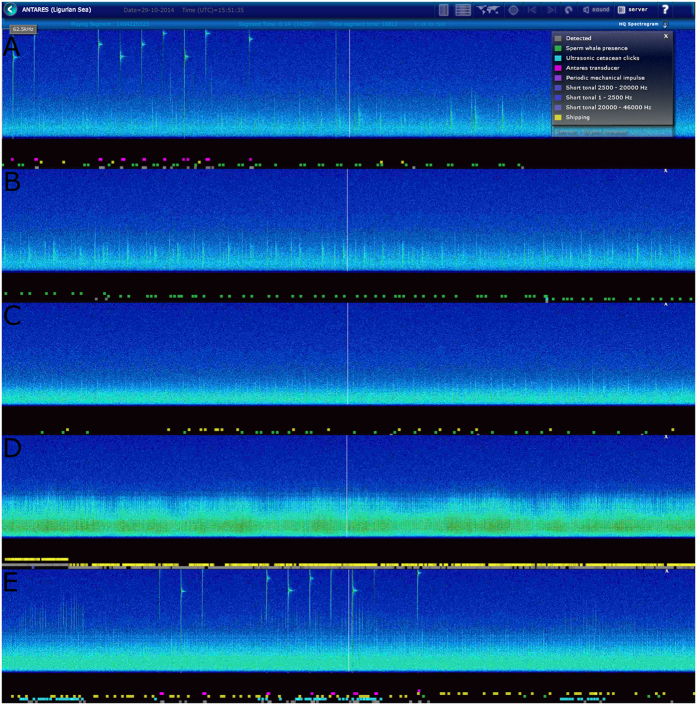
Series of screenshots from http://listentothedeep.com interface where the output of the classification process is displayed. The legend in A shows coloured squares that are attributed to each class of detected signals displayed underneath the spectogrammes: green for SWC (sperm whales), yellow for ISC (shipping), blue for UCC (cetacean sonar), pink for the ANTARES localisation mechanical pings. (**A**) Sperm whales are detected and classified as well as the ANTARES transducer pings. (**B**) Presence of a group of sperm whales. (**C**) Sperm whales in presence of incoming ship noise. (**D**) Ship noise is masking the low frequency part of the spectrogramme. (**E**) SWC, UCC, ISC and ANTARES transducer pings.

The classification modules used in this article were designed to operate under such conditions; they are part of a larger system^11^, which includes impulse and short tonal sound detectors and classifiers applied to several frequency bands covering the whole bandwidth of the data. The modules work on segments (~16.8 seconds with a sampling rate of 250 kHz) of the audio stream that are automatically displayed in real-time on the http://listentothedeep.com interface (Fig. 6).

In this paper, we concentrate on the sperm whale impulse classification^32^ (SWC) and its performance in the presence of other impulsive sounds that were classified like ultrasonic cetacean impulses (UCC) and impulsive shipping noise (ISC). Ultrasonic cetacean impulses refer to other cetacean species vocalisations, in particular Delphinid and Zifiid echolocation sounds whose lower frequencies overlap with the sperm whale frequency range. Other impulsive sources overlapping with the sperm whale frequency range were found to be very rare at the ANTARES platform.

Each detected impulse was systematically processed with a feature extraction step^32^ and assigned a class with a classifier based on Gaussian Mixture Models (GMM). After classification, each detected impulse was tagged as SWC, ISC, or UCC. Impulses that were not recognized by the classifier received no tag and their effect on subsequent processing was neutral.

False positive rates - proportion of positives incorrectly classified - and false negative rates - proportion of positives not detected or detected but incorrectly classified - were estimated for different thresholds in the three classes (see Table 4).

The number of impulses assigned to each class per segment (the *detection rate*) was recorded; it is abbreviated SWI for the sperm whale class, ISI for the impulsive shipping class, and UCI for the ultrasonic cetacean class. The sources of these classes tended to produce long impulse trains covering multiple segments and providing reasonably continuous detection time intervals. For sperm whales this situation was different when only a single animal was present or when the animals were far away from the hydrophone. The number of detected impulses in a segment could differ considerably between consecutive segments. In some cases, a segment had zero detections while neighbouring segments did show sperm whale impulses. To obtain a smoother detection rate for sperm whale impulses (SWI) a running weighted average was computed over the number of impulses in a segment that were classified as sperm whale (SWC), as demonstrated in the paper by Zaugg *et al*.^32^. Thus SWI for a segment *i* was given by (1):


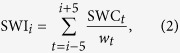


where w_t_ = 5 if *t* = *i* and 1 otherwise, effectively evaluating the presence of sperm whale impulses in a 3 minute time interval^32^. The same was done for ultrasonic and shipping impulses.

In addition to the detections, noise levels were measured as well, calculating the sound pressure level (SPL) in each segment (2):


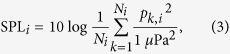


where *p*_*k,i*_ is the measured pressure in sample *k* and *N*_*i*_ is the number of samples in a segment *i*.

The computed level does not represent the true sound pressure level at the site because the hydrophones of the AMADEUS system operate a high pass filter at 2 kHz. Since there is no interest here in absolute sound levels, but only in knowing when high levels of sound were present that might mask sperm whale signals or otherwise affect the classification system, the SPL (Sound Pressure Levels) reported in this paper are relative to the median SPL calculated over all available segments in 2012 (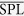).

### Statistical analysis of the LIDO classifications

In this study, we aim at presenting an analysis of sperm whale abundance at two temporal scales (hours and months), and how shipping can influence the sperm whale detection rates in both scales.

We define sperm whale acoustic presence (SWP) as a binary variable whose value is 1 if a human specialist is able to identify a sperm whale impulse registered with the AMADEUS system in a user-defined interval of time (in this case, in a segment of 16.8 s, see Zaugg *et al*.^32^). Its value is 0 otherwise. Shipping presence (ISP), expressed as ship clicks, is also defined with the same strategy. Presence is to be understood as presence within the detection range of the observatory. This assignment by a human specialist is a gold standard commonly used to calibrate automated methods. From this point of view, we modelled SWP and ISP as a function of the information provided by the LIDO classification process. This enabled us to automatically predict presence for new segments and correct for possible false positive and false negative impulse situations.

To fit this model we constructed a training data set with 963 segments spread over all hours of the day (at least 30 observations per hour). Initially, 400 segments of 16.8 s were randomly selected from 625 recordings of approximately 67 s (4 segments) each, which were stored every 7 hours from June 2011 to December 2011. Furthermore, 563 segments were added to the data set. These were randomly selected from 490 recordings of >5 min (20 segments) each, which were stored every 2 hours from January 2013 to March 2013. Experienced observers analysed every segment to decide whether sperm whale impulses and impulse shipping noise were present or not (i.e. they assigned SWP and ISP to those segments). Here, we assumed that the environment conditions found in this data were representative of what occurred in other segments, which were not in the training data regardless of the hour, day or month.

We considered a generalised linear model with a binomial response and a logit link function (GLM, Nelder 1972^33^) to predict sperm whale presence (SWP = 0: absence; SWP = 1: presence) as a function of SWI and ISI, including the interaction between the two. A logarithmic transformation was applied both to SWI and to ISI (X’ = log[X + 1]). Above a certain threshold the exact value of SWI/ISI is not very important and taking the logarithm reduces the range of the parameters. Similarly, to predict the presence of shipping impulses in a segment (ISP = 0: absence; ISP = 1: presence), a generalised linear model in R was fitted taking into account the information in ISI, SWI and UCI. The same logarithmic transformation used for the first model was applied to the three independent variables. Both models were fitted in R version 2.15.2 (R Core Team 2012). The prediction of the GLM model is a continuous value between 0 and 1, which can be interpreted as an index of sperm whale acoustic presence.

The main objective of this study was a reliable prediction of sperm whale (acoustic) presence during a particular hour of the day. To make an hourly prediction, SWP was summed over the segments that were analysed during that hour, i.e. a percentage of segments with SWP. In order to obtain a measure of how well the model predicts sperm whale presence, a bootstrap approach was applied to compute a confidence interval around SWP. A thousand bootstrap resamples (drawn with replacement, same size = 963 segments) were obtained from the training data. The model fitted to each of these data sets was then used to estimate SWP on the original training data (summing SWP over all segments).

Sperm whale presence and impulse ship presence predictions were aggregated by month and by hour. Confidence intervals were found using 500 bootstrap samples.

Moreover, SWP predictions in each month were separated in two groups of hours: daylight segments [6 to 18 h) and night segments [18 to 6 h). A Wilcoxon paired test of size 12 (the number of months) was used to determine whether significant differences between the two groups were observed.

### Sperm whale presence predictions as function of SPL (noise level)

The detector reliability was generally weaker when high SPL were found in the recordings. This effect can be caused by a sound source that completely covered the sonar detection band with continuous noise. This phenomenon is called masking. However, a high SPL does not always indicate that sonar was masked. Due to the nature of the SPL computation, a single high-level transient signal can produce a high SPL output computed over a segment. At ANTARES this happened quite often because the neutrino detection lines are calibrated using high-frequency high-level echo sounders that transmit a sequence of pings about every once 100 s (see Fig. 6A and E). On these occasions, there would be no masking of sonar if no other continuous source was present. Segments that contained such a ping were easily identified due to their particular acoustic signature.

In order to obtain more information on the sperm whale behaviour and to see how the SPL affected the daily distribution of SWP, an overview was made showing SWP during each hour of the day and for increasing SPL (shown relative to the yearly median 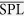).

## Additional Information

**How to cite this article**: André, M. *et al*. Sperm whale long-range echolocation sounds revealed by ANTARES, a deep-sea neutrino telescope. *Sci. Rep.* **7**, 45517; doi: 10.1038/srep45517 (2017).

**Publisher's note:** Springer Nature remains neutral with regard to jurisdictional claims in published maps and institutional affiliations.

## Figures and Tables

**Table 1:** Sperm whale acoustic presence model: estimated coefficients, standard errors on the coefficients, corresponding Z value and two-sides probability.

Coefficient	Estimation	Std. Error	Z value	Pr (>|z|)
Intercept	−3.956	0.303	−13.057	<2e-16
SWI’	3.645	0.355	10.286	<2e-16
ISI’	−0.073	0.153	−0.477	0.6332
SWI’ * ISI’	−0.379	0.116	−3.264	0.001

Intercept, SWI’ and the interaction SWI’*ISI’ were significant. ISI’ by itself was not significant.

**Table 2:** Impulsive ship noise presence model coefficients: estimated coefficients, standard errors on the coefficients, corresponding Z value and two-sides probability.

Coefficient	Estimation	Std. Error	Z value	Pr (>|z|)
Intercept	−1.949	0.199	−9.774	<2e-16
ISI’	2.636	0.174	15.121	<2e-16
UCI’	−0.444	0.062	−7.191	2e-13
SWI’	0.028	0.233	0.121	0.903
SWI’ * ISI’	−0.627	0.095	−6.586	4e-11

Intercept, ISI’, UCI’ and interaction SWI’ * ISI’ were significant. SWI’ by itself was not significant.

**Table 3:** Source presence estimated values during 2012: total number of segments with sperm whale and shipping impulse presence by month.

Month	Jan	Feb	Mar	Apr	May	Jun	Jul	Aug	Sep	Oct	Nov	Dec
SWP	36763	14207	27447	10452	12210	12969	41661	28112	27700	19363	22672	18269
ISP	42294	34262	42445	36166	47073	48394	52335	57071	48622	40194	48833	33399
Total	159428	149142	159428	154285	159428	154285	159428	159428	154285	159428	154285	159428

The bottom row shows the number of 16.8 second segments per month.

**Table 4:** Training data accuracy: threshold (λ), area under the curve (AUC), false positive rates (FPR), false negative rates (FNR), negative predictive value (NPV) and positive predictive value (PPV) from sperm whales, shipping and dolphins presence.

Statistic	λ	AUC	FPR	FNR	NPV	PPV
SWC	3	0.84 (0.78–0.90)	0.03 (0.02–0.05)	0.42 (0.30–0.54)	0.93 (0.92–0.95)	0.75 (0.64–0.85)
ISC	5	0.93 (0.90–0.96)	0.09 (0.06–0.13)	0.14 (0.08–0.20)	0.93 (0.90–0.95)	0.82 (0.77–0.87)
UCC	9	0.93 (0.89–0.96)	0.14 (0.08–0.21)	0.07 (0.04–0.10)	0.83 (0.77–0.88)	0.94 (0.92–0.96)

Confidence intervals are at 95%, with the AUC, FPR and FNR calculations using 2,000 stratified bootstrap replicates and the NPV and PPV for the asymptotic limits.
